# Simultaneous determination of nine phenolic compounds in imitation wild *Dendrobium officinale* samples using ultrahigh-performance liquid chromatography–tandem mass spectrometry

**DOI:** 10.3389/fnut.2023.1129953

**Published:** 2023-04-13

**Authors:** Yingsu Mu, Li Cheng, Xiaojian Gong, Jiangxiong Ma, Shiyu Zhang, Yinghua Mu, Kang Liang, Xin Zhou, Chao Zhao

**Affiliations:** ^1^Key Laboratory for Information System of Mountainous Areas and Protection of Ecological Environment, Guizhou Normal University, Guiyang, China; ^2^Guizhou Engineering Laboratory for Quality Control and Evaluation Technology of Medicine, Guizhou Normal University, Guiyang, China; ^3^Guizhou University of Traditional Chinese Medicine, Guiyang, China; ^4^College of Food and Pharmaceutical Sciences, Ningbo University, Ningbo, China; ^5^Guizhou Forestry Scientific Research Institute, Guiyang, China

**Keywords:** *Dendrobium officinale*, phenolic components, UHPLC-QQQ-MS/MS, provenance, method validation

## Abstract

*Dendrobium officinale* Kimura et Migo (*D. officinale*), one of the nine everlasting types of grass, has gained increasing attention owing to its important roles in alternative medicines and drug discovery. Due to its natural resources being in danger of being extinct, imitation wild planting is becoming increasingly common. To assess the product’s quality completely, an efficient ultrahigh performance liquid chromatography-triple quadrupole tandem mass spectrometry (UHPLC-QQQ-MS/MS) method was established to simultaneously quantify nine phenolic compounds in *D. officinale* samples. The extraction parameters, including solvent, solvent concentration, solid–liquid ratio, and extraction time, were systematically optimized with the single-factor test. The results demonstrated that extraction with a 1:200 solid-to-liquid ratio of 80% methanol for 1.5 h was the most efficient condition for the extraction of flavonoids. Satisfactory retention times and resolution of the nine analytes were acquired on the Thermo Scientific Hypersil GOLD column with multiple reaction monitoring in negative ion scanning mode. The method was validated to demonstrate its selectivity, linearity, precision, accuracy, and robustness. Thus, the verified UHPLC-QQQ-MS/MS method was successfully applied to the quantification of phenolic components present in *D. officinale* samples. The results indicated that the quantity and composition of phenolic components in *D. officinale* from various provenances were significantly different. This work provides a theoretical foundation for the cultivation and assessment of wild *D. officinale* quality.

## 1. Introduction

*Dendrobium officinale* Kimura et Migo (*D. officinale*), a perennial epiphytic member of the Orchidaceae, is considered to have the best medicinal properties in traditional Chinese medicine (1, 2). *Dendrobium officinale*, is a well-known medicinal and food homologous plant, that strengthens the stomach and promotes the production of body fluid, nourishing Yin and clearing heat (3, 4). It is known that *D. officinale* is primarily distributed in several nations, including the United States, Japan, and Australia, and is more broadly distributed in southern China, including the provinces of Zhejiang, Anhui, Fujian, Guangxi, Yunnan, and Guizhou (3, 5). However, *D. officinale* has strict requirements for habitat conditions, a slow growth rate, low yield, and excessive harvesting, which has led to a sharp decrease in the number of wild plants and has been included in the “*China Plant Red Data Book*” (6). Currently, the market for *D. officinale* mainly comes from greenhouse cultivation and imitation wild planting, among which imitation wild planting improves the quality of *D officinale* while making full use of woodland resources, with low cultivation cost and a good ecological environment. However, the 2020 edition of the Chinese Pharmacopoeia only uses polysaccharide content as its quality evaluation index, which is contrary to the theory that complex components in Chinese medicine interact with each other and work synergistically. Therefore, we should comprehensively analyze the active ingredients of *D officinale* and establish a more scientific quality control standard for *D officinale* (7).

Phenolic compounds include flavonoids, simple phenols and quinones. Numerous natural phenols have attracted great interest from scientists around the world because they are considered safer and have a wide range of health-promoting properties. Flavonoids are a widespread group of secondary metabolites in plants that not only play a key role in the pharmaceutical industry but also serve as excellent chemical markers for quality control of medicinal plants (8). *Dendrobium officinale*’s active pharmaceutical ingredients include phenols, flavonoids, alkaloids, amino acids, coumarins, terpenes, benzylic compounds, and several trace minerals, in addition to polysaccharides (9–12), which have been widely used to treat hyperglycemia (13), hyperlipidemia (14), and immune enhancement (15) and to benefit the stomach (16). Polysaccharides are the predominant bioactive compounds in these substances. Of course, phenolic components are a group of compounds that are also prevalent in *D. officinale*. In recent years, interest has increased due to the potent antioxidant and protective effects of phenolic components against cell toxicity (17). Phenolic contents have been effectively isolated, and their structures have been validated. Unfortunately, the development of phenolic components from *D. officinale* has been hampered by the lack of a reliable method for quantitative determination. With the birth and development of large-scale instruments and equipment such as gas chromatographs (GC), liquid chromatography (LC), ultrahigh-performance liquid chromatography (HPLC), and mass spectrometers (MS), instrumental analysis methods have become the most commonly used methods for quantitative analysis of secondary metabolites of plants (18–20). Zhu et al. (21) used an HPLC assay to simultaneously quantify 11 phenolics in four *Dendrobium* species. The contents of kaempferol, quercetin and myricetin in flavonoids are mainly determined based on the HPLC method (22). However, some phenolic components cannot be effectively identified and quantified using HPLC because of poor separation from more abundant components (23). Compared with ultrahigh-performance liquid chromatography (HPLC), ultrahigh-performance liquid chromatography–tandem mass spectrometry (UHPLC–MS/MS) has the characteristics of higher-resolution separation and better identification and quantification of individual components (19, 24–27).

To establish the method of UHPLC-QQQ–MS/MS for the first time for the simultaneous determination of various components of phenolic *D. officinale* for overall quality control. The extraction conditions were optimized, and the extraction method, extraction solvent, solvent concentration, extraction time, and material-to-liquid ratio were investigated. The contents of ferulic acid, chrysin, naringenin, luteolin, L-epicatechin, quercetin, isorhamnetin, cynaroside, and naringin in *D. officinale* from different provenances were determined, and the quality of nine phenolic compounds was comprehensively evaluated by the TOPSIS comprehensive evaluation method. This study may offer a workable and straightforward technique for quality control of imitation wild *D. officinale* as well as a theoretical framework for its development and cultivation.

## 2. Materials and methods

### 2.1. Instruments, reagents, and materials

The TSQ Quantum Liquid Chromatography–Mass Spectrometer (UHPLC–MS/MS) included a triple quadrupole mass analyzer, ESI ion source, and Xcalibur workstation. The liquid phase part was a Thermo Accela UHPLC, including an Accela PDA detector, Accela autosampler and Accela 1250 infusion pump, which were purchased from Thermo Fisher Scientific, Inc. XS-105DU 1/100000 and the AL204 1/10000 electronic analytical as well as the KQ-5200E type ultrasonic cleaner were both obtained from Kunshan Ultrasonic Instruments Co.

**Figure 1:**
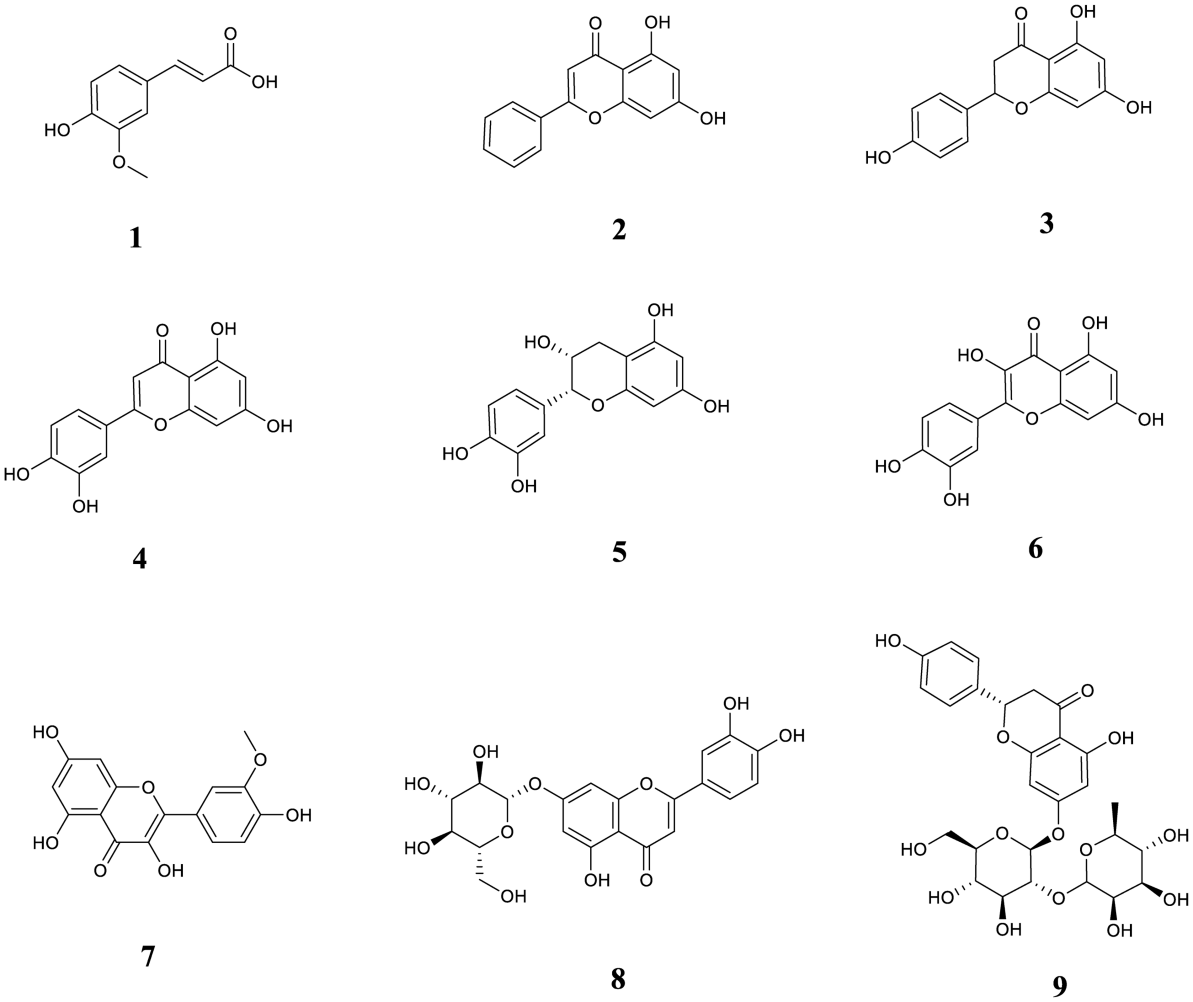
The chemical structures of nine phenolic components in this study. **(1)** ferulic acid; **(2)** chrysin; **(3)** naringenin; **(4)** luteolin; **(5)** L-epicatechin; **(6)** quercetin; **(7)** isorhamnetin; **(8)** cynaroside; **(9)** naringin.

Quercetin was purchased from the China Institute of Food and Drug Control; Ferulic acid and L-epicatechin were purchased from the China Institute for Testing and Certification of Pharmaceutical and Biological Products. Chrysin, naringenin, luteolin, and cynaroside were acquired from Guizhou Dida Biotechnology Co., Ltd. Isorhamnetin was acquired from Chengdu Pfeiffer Biotechnology Co., Ltd. Quercetin was acquired from the China Institute of Food. Naringin was purchased from Chengdu Botanical Standard Pure Biotechnology Co., Ltd., Research Central Reference Materials Research Center. The purities of the 9 reference substances were all greater than 98%. The analytical purities of methanol (MeOH) and ethanol were purchased from Tianjin Comitry Co., Ltd. Acetonitrile (HPLC grade) was purchased from American TEDIA Company. Formic acid (LC/MS grade) was acquired by ROE SCIENTIFIC Corporation, United States. Food-grade distilled water was purchased from Watson’s Food and Beverage Guangzhou Co. The structural formulas of the 9 compounds are shown in Figure 1.

The majority of medicinal plants are harvested and grown in Yun Guan Shan, a state-owned forest farm in Guizhou Province, China, where they mimic wild *D. officinale* from *Pinus massoniana Lamb.* tree species.

### 2.2. Sample pretreatment and standard solution

All dry ingredients were ground up and put through a sieve with a mesh size of 60. As mentioned below, 0.1 g of samples were weighed exactly (within 0.00001), and they were then extracted once with 80% methanol (20 ml) by reflux for 1.5 h. The sample was meticulously weighed before extraction, and the weight loss was compensated once the sample solution was weighed and cooled to room temperature. All of the solutions were filtered through a 0.22 μm microporous membrane before being put on the device.

Each standard was properly weighed. The reference standards’ standard stock solutions were made by combining and evaporating in 80% methanol. The working standard solution was then made by gradient-diluting the standard stock solution with the same solvent, and both of these solutions were stored at 4°C for further analysis.

### 2.3. Instrumentation and chromatography

#### 2.3.1. Ultrahigh-performance liquid chromatography conditions

The chromatographic separations were performed on a thermostat-controlled, 25°C, Thermo Scientific Hypersil GOLD column (50 mm × 2.1 mm, 5 μm). The following procedure was used for the gradient elution of 0.1% formic acid acetonitrile (solvent A) and 0.1% formic acid aqueous solution (solvent B) at a flow rate of 200 μl/min: 0–3 min, 5% A; 3–3.1 min, 5–80% A; 3.1–6 min, 80% A; 6.0–6.1 min, 80–5% A and 6.1–12 min, 5% A. The injection volume was 10 μl.

#### 2.3.2. Mass spectrometer conditions

**Figure 2:**
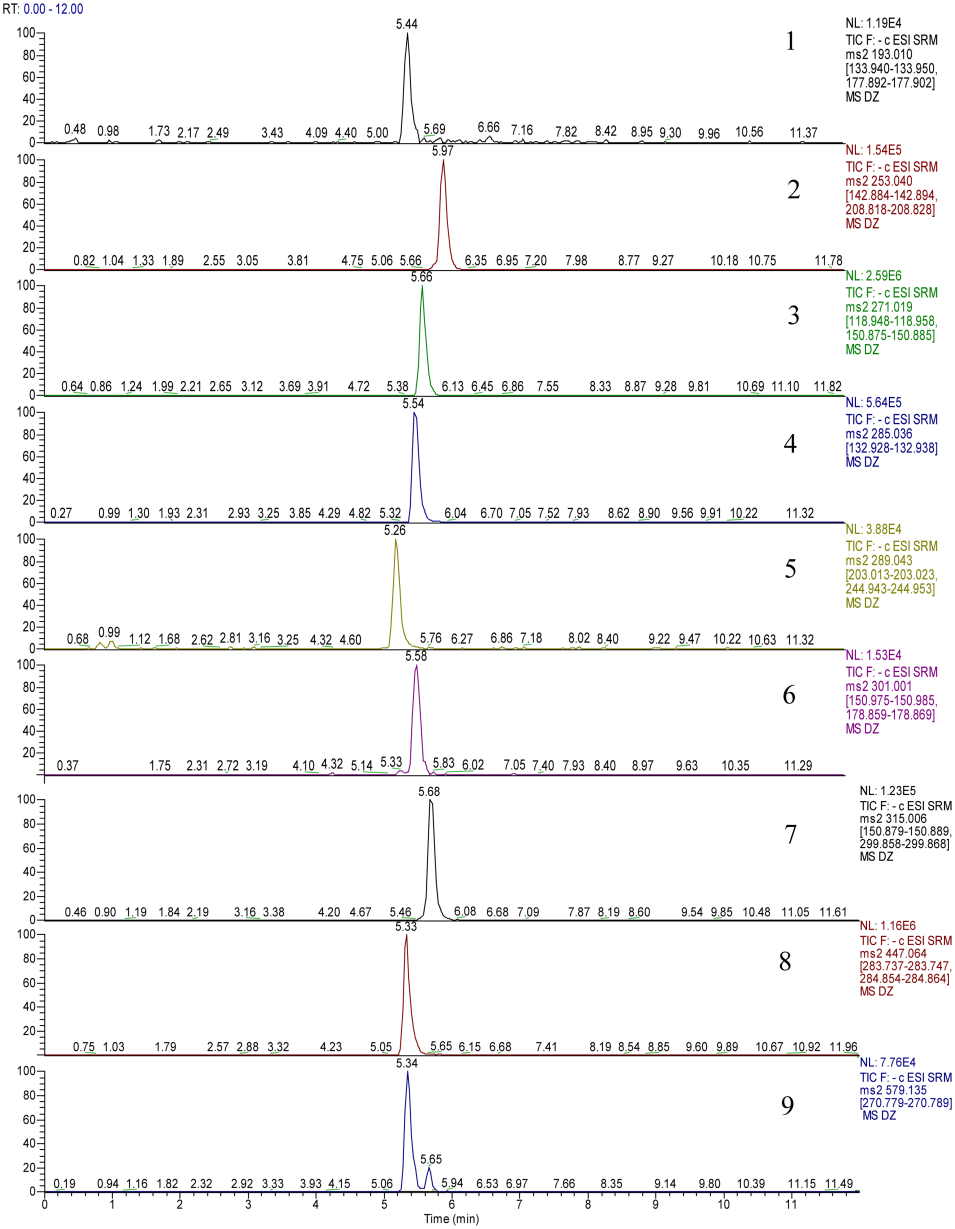
Multiple reaction monitoring (MRM) chromatograms of 9 components.

Electrospray ionization in the negative ionization mode was used to obtain the mass spectra. The ESI source performed best under the following conditions: spray voltage of 2,500 V, the capillary temperature of 350°C, vaporizer temperature of 200°C, sheath gas pressure of 35 Arb, the auxiliary gas pressure of 15 Arb, and vaporizer temperature of 200°C. Multiple reaction monitoring (MRM) was used as the measurement technique, and 0.1 s was the scanning interval. Table 1 displays the precise quantitative analysis. Figure 2 displays 9 compounds’ MRM diagrams.

**Table 1:** Mass spectral parameters of the nine components.

Compound	Molecular weight	Chemical formula	Parent mass (m/z)	Product mass (m/z)	T Lens (v)	Collision voltage (m Torr)	Collision energy (eV)
ferulic acid	194.18	C10H10O4	193.010	133.945	73	1.5	18
				177.897	73	1.5	14
Chrysin	254.24	C15H10O4	253.040	142.948	120	1.5	26
				185.062	120	1.5	21
Naringenin	272.25	C15H12O5	271.019	118.953	107	1.5	30
				150.880	107	1.5	21
Luteolin	286.05	C15H10O6	285.036	132.933	123	1.5	36
L-Epicatechin	290.27	C15H14O6	289.043	203.018	130	1.5	14
				244.948	130	1.5	12
Quercetin	302.24	C15H10O7	301.001	150.980	109	1.5	19
				178.864	109	1.5	12
Isorhamnetin	316.26	C16H12O7	315.006	150.884	116	1.5	20
				299.863	116	1.5	15
Cynaroside	448.38	C21H20O11	447.064	283.742	133	1.5	40
				284.859	133	1.5	29
Naringin	580.18	C27H32O14	579.135	270.784	182	1.5	33

### 2.4. Extraction optimization

Different extraction methods were investigated by a single-factor experiment: ultrasound (100 W, 90 kHz, 60 min), reflux (60 min, 67°C), and cold soaking (60 min); different solvents: ethanol, methanol, water, different concentrations: methanol (40, 60, 80, and 100%). Then the effects of different solid–liquid ratios of 1:200, 1:300, 1:400, and 1:500 (*m*/*v*), and reflux extraction times of 0.5, 1, 1.5, and 2 h on the extraction were studied. The supernatant was examined by “2.3” chromatography after being filtered using a 0.22 μm microporous organic membrane. Nine peaks’ response values, peak areas, and peak forms were compared.

### 2.5. Method validation for quantitative analysis

#### 2.5.1. Linearity LOQ and LOD

The master batch of the control solution was aspirated and prepared into a mass concentration gradient solution. Following “2.3″‘s chromatographic conditions, the mass concentration was employed as the horizontal coordinate (*X*), and the peak area as the vertical coordinate (*Y*). The standard curve’s lowest concentration point is called the LOQ. Signal-to-noise ratios (S/N) of 3 were used to calculate the limits of detection (LOD).

#### 2.5.2. Precision, stability, repeatability, and recovery

The intraday and interday variations were used to investigate the accuracy of the suggested approach. The intraday precision was calculated by following the standard curve for three consecutive days, mixing 9 standards, injecting them six times, and calculating the concentration and relative standard deviation (RSD%) at each level. At various time intervals of 0, 2, 4, 8, 12, and 24 h, the sample was injected and examined. Six identical samples were prepared in parallel to test the repeatability of the process. Three different concentration levels of the standard solutions—50, 100, and 150%—were added to the sample powder for the recovery test to assess the method’s accuracy. The sample powder was then extracted, and the results were examined. For each level, three parallel samples were taken.

### 2.6. The Technique for Order Preference by Similarity to Ideal Solution evaluation

The Technique for Order Preference by Similarity to Ideal Solution (TOPSIS) method is a multi-index decision analysis method, that calculates the multi-index as a comprehensive index, and transforms the multidimensional problem into a one-dimensional problem, which greatly reduces the interference of different types of indicators on decision-making in the analysis process, and significantly improves the scientificity and accuracy of multiobjective decision analysis (28). It has been widely used in the quality evaluation of various Chinese medicinal materials.

## 3. Results and discussion

### 3.1. Qualitative analysis

**Figure 3:**
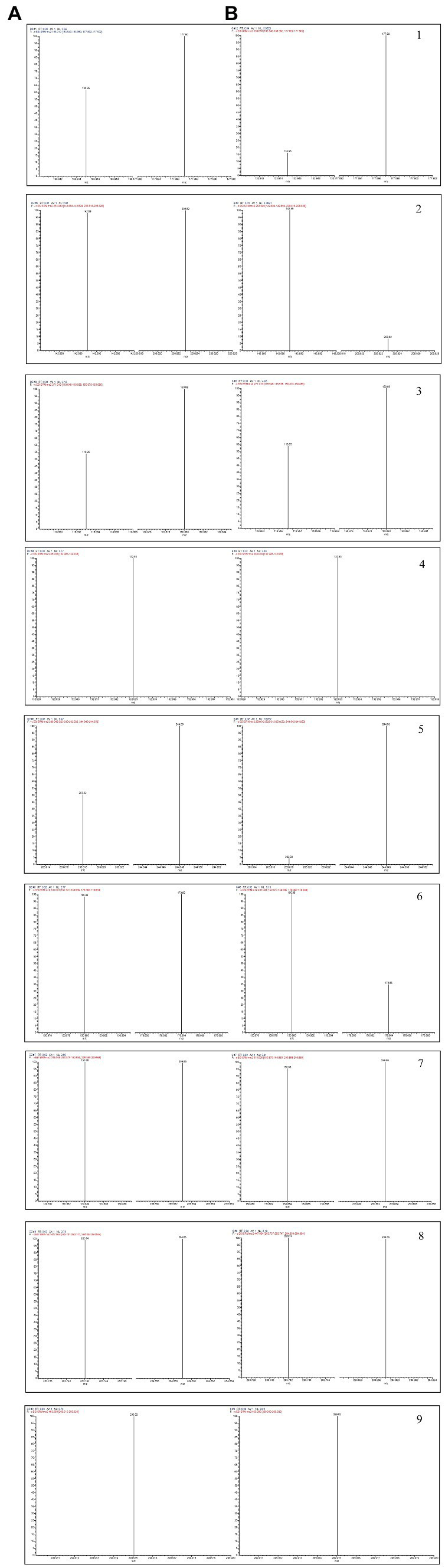
The tandem mass spectrometry (MS/MS) spectra of the standards **(A)** and the phenolic components in the samples **(B)**.

In this paper, 597 metabolites were detected based on the UHPLC–MS/MS detection platform and a self-built database, and 185 flavonoids were screened through primary classification. This part of the experiment was performed by Wuhan Maitville Biotechnology Co., Ltd. Therefore, the UHPLC–ESI MS/MS method was established to compare the total ion current diagram and fragment ion diagram of the *D officinale* extract and standard sample, and nine phenolic compounds were identified, ferulic acid (1), chrysin (2), naringenin (3), luteolin (4), L-epicatechin (5), quercetin (6), isorhamnetin (7), cynaroside (8), and naringin (9), as shown in Figure 3.

### 3.2. Extraction optimization

**Figure 4:**
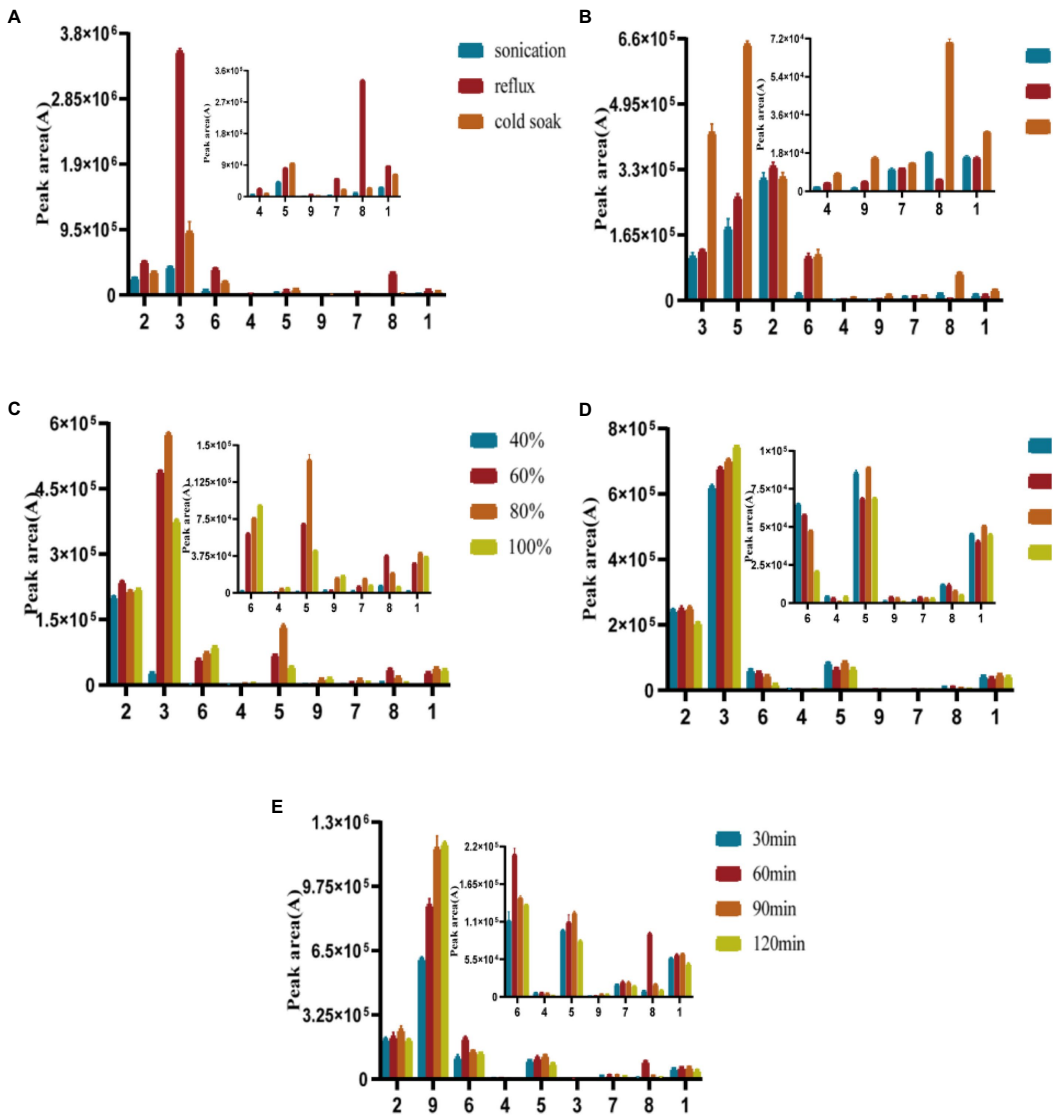
Optimization of extraction methods **(A)**, extraction solvent **(B)**, solvent concentrations **(C)**, extraction stock ratios **(D)**, and extraction times**(E)**.

The following extraction methods (sonication, reflux, cold soak), extraction solvents (ethanol, methanol, water), solvent concentrations (40, 60, 80, 100% methanol), extraction stock ratios [1:200, 1:300, 1:400, 1:500 (m/v)], and extraction times (0.5, 1, 1.5, and 2 h) were optimized, as shown in Figure 4. Reflux extraction was chosen for the herb’s extraction because it produced much more naringenin and cynaroside than the other two extraction techniques. More phenols were extracted from methanol, while more polysaccharides were extracted from aqueous extracts that were not well filtered. The longer the extraction time was, the greater the extracted phenol content increased, but when the time was 1.5 h after the slow growth. The extraction of phenolic compounds was not significantly impacted by the material-to-liquid ratio. The feed-to-liquid ratio of 1:200 and 1.5 h of refluxing 80% methanol resulted in the best extraction conditions.

### 3.3. Validation of the quantitative analysis method

The mass concentration was used as the horizontal coordinate (*X*), and the peak area was used as the vertical coordinate in the linear regression (*Y*). The regression equation and correlation coefficient (*R*^2^) were calculated, and the maximum LOD and LOQ of the 9 compounds were 16.89 and 15 ng/ml, respectively. The result shown in Table 2, show good linearity.

**Table 2:** Linear regression date of nine components.

Compound	Regression equation	*R* ^2^	Linearity range (ng/mL)	LOD (ng/mL)	LOQ (ng/mL)
Ferulic acid	*Y* = 208.33 *X* + 1515.2	0.9992	15 ~ 1,500	15	9.11
Chrysin	*Y* = 6,986 *X* + 106,987	0.9992	5 ~ 500	5	1.77
Naringenin	*Y* = 22,034 *X* + 320,994	0.9991	8 ~ 800	8	8.96
Luteolin	*Y* = 18,117 *X* + 14,977	0.9993	1 ~ 100	1	0.27
L-Epicatechin	*Y* = 1397.4 *X* + 17,484	0.999	14 ~ 1,400	14	16.89
Quercetin	*Y* = 6877.6 *X* + 1,084	0.9992	3 ~ 300	3	3.96
Isorhamnetin	*Y* = 26,369 *X* + 12,225	0.9994	1 ~ 100	1	0.49
Cynaroside	*Y* = 28,092 *X*–7772.9	0.9993	1 ~ 100	1	0.37
Naringin	*Y* = 18,604 *X*–835.32	0.9992	0.05 ~ 5	0.05	0.05

The within-day and between-day variations were used to evaluate the precision of this UHPLC–MS/MS method for measuring *D. officinale*. The relative standard deviation RSD for the intraday and interday precision ranged from 1.60 to 7.49% and from 5.89 to 9.99%, respectively. Then, the relative standard deviation for repeatability and stability varies from 1.49 to 12.50%. Table 3 presents the outcomes. According to Table 4, the average recoveries of the spiked trials ranged from 79.87 to 99.15%, with RSDs between 7.02 and 16.98%. The experimental outcomes further showed that the analytical approach is reliable and capable of fulfilling the assay’s requirements.

**Table 3:** Stability, repeatability, inter-day precision, and intra-day precision of nine components.

Compound	Stability		Repeatability		Precision			
					Intra-day		Inter-day	
	Mean	RSD (%)	Mean	RSD (%)	Mean	RSD (%)	Mean	RSD (%)
Ferulic Acid	380.18	1.49	46.60	7.21	47.77	4.61	417.29	9.99
Chrysin	19.43	5.65	42.51	7.36	48.90	2.16	14.79	7.11
Naringenin	115.94	4.23	49.82	6.42	57.48	4.31	114.45	7.83
Luteolin	0.41	9.83	11.84	4.04	7.37	1.60	0.58	7.82
L-Epicatechin	85.02	3.89	110.64	3.41	114.43	2.40	60.49	6.02
Quercetin	38.76	3.56	18.42	6.43	17.14	2.35	25.45	9.85
Isorhamnetin	2.54	8.49	6.46	2.92	6.45	4.87	0.36	8.21
Cynaroside	1.09	3.18	7.59	1.53	7.31	6.58	1.26	6.55
Naringin	0.49	7.14	0.78	12.50	0.66	5.17	20.49	6.35

**Table 4:** Results of *Dendrobium officinale* spiked sample recovery experiment.

Compound	Average recovery (%)	RSD (%)	Compound	Average recovery (%)	RSD (%)
Ferulic Acid	93.39	7.02	Quercetin	86.97	14.83
Chrysin	89.17	14.94	Isorhamnetin	89.43	12.07
Naringenin	87.25	11.68	Cynaroside	86.07	16.98
Luteolin	79.87	7.47	Naringin	86.79	14.80
L-Epicatechin	90.20	10.91			

### 3.4. *Dendrobium officinale* sample analysis

The validated method is used to analyze samples from different sources. Table 5 displays the quantitative results obtained from the calibration curve. Each sample was analyzed in triplicate. A total of 9 flavonoids were detected in the *D officinale* samples. The ferulic acid, naringenin, and epicatechin contents were higher in each provenance than the other components, while the luteolin content was lower in each provenance. Of these, the Guangxi provenance had the highest levels of ferulic acid and L-epicatechin, at 88.27 and 109.68 μg/g, respectively. Chrysin, naringenin, quercetin, and isorhamnetin were all much more abundant in ZJ origin than they were in other compounds, while luteolin and naringenin were the least abundant of all ZJ provenances. The impacts of geographical origins and storage conditions may account for the significant variations in flavonoid composition and content between batches of *D. officinale* samples.

**Table 5:** The content of nine compounds in *Dendrobium officinale* samples (μg/g).

Compound	FJ	AL	FJS	GX	ZJ
Ferulic acid	77.85 ± 2.60	71.00 ± 5.68	31.06 ± 4.55	88.27 ± 1.96	55.36 ± 3.52
Chrysin	2.14 ± 0.73	2.56 ± 0.98	2.62 ± 0.62	2.33 ± 0.06	10.20 ± 0.46
Naringenin	60.18 ± 0.34	35.74 ± 1.28	67.07 ± 0.58	67.96 ± 0.09	76.56 ± 1.66
Luteolin	0.60 ± 0.46	0.12 ± 0.07	0.19 ± 0.04	0.30 ± 0.03	0.20 ± 0.10
L-Epicatechin	37.28 ± 1.07	101.09 ± 2.62	94.86 ± 9.55	109.68 ± 2.87	67.54 ± 4.52
Quercetin	3.16 ± 0.05	2.13 ± 0.32	2.31 ± 0.25	2.44 ± 0.24	4.84 ± 0.40
Isorhamnetin	0.32 ± 0.05	0.23 ± 0.02	0.30 ± 0.11	0.23 ± 0.02	0.80 ± 0.40
Cynaroside	0.39 ± 0.09	0.35 ± 0.04	0.31 ± 0.02	0.42 ± 0.08	0.42 ± 0.20
Naringin	0.11 ± 0.02	0.24 ± 0.00	0.21 ± 0.06	0.14 ± 0.06	0.20 ± 0.20

The quality of imitation wild *D. officinale* was properly evaluated by TOPSIS, and elevated provenances were selected based on their phenol content. *D* + is the distance between each treatment and the optimal index, and *D* − is the distance from the worst vector. The smaller the value is, the closer it is to the optimal index or the worst index. CI = *D*–/(*D* + + *D*–), the greater the value, the better the overall efficiency. According to the CI value of each index, the advantages and disadvantages of the 5 provenances were as follows: Zhejiang > Fujian > Guangxi > Anlong > Fanjingshan. Among them, the Anlong, Fanjingshan, and Guangxi CI values are close to the specific data shown in Table 6. The maximum CI of the ZJ provenance is 0.7296, which can be further studied and developed as a superior quality provenance (Table 6).

## 4. Conclusion

In this study, an effective UHPLC-QQQ-MS/MS technique for the isolation and quantification of nine phenolic compounds in *D. officinale* was effectively established and verified. Following optimization, samples were extracted using reflux and an aqueous solution of 80% methanol. The UHPLC-QQQ-MS/MS technology developed in the current study is precise and sensitive for quantifying the main phenolic components. Further analysis with TOPSIS using the contents of the nine phenolic compounds suggested that *D. officinale* should be screened for high-quality ZJ seed sources. The validation data demonstrated satisfactory linearity, precision, accuracy, repeatability, and stability. According to the findings, the UHPLC-QQQ-MS/MS approach shows excellent potential for use in the investigation of bioactive substances in herbal medicines.

**Table 6:** The Technique for Order Preference by Similarity to Ideal Solution (TOPSIS) evaluation results (7).

Index value	(*D*+)	(*D*–)	CI	Rank
FJ	0.6964	0.3942	0.3614	2
AL	0.8111	0.2570	0.2406	4
FJS	0.7695	0.2248	0.2261	5
GX	0.7582	0.3174	0.2951	3
ZJ	0.2933	0.7914	0.7296	1

## Data availability statement

The original contributions presented in the study are included in the article/supplementary material, further inquiries can be directed to the corresponding authors.

## Author contributions

YsM, LC, and XG finished all the experiments. CZ and XZ contributed to the concept development and outline arrangement, and revised the work critically for important intellectual content. YhM and SZ contributed to relevant references by collecting and drawing pictures. JM and KL analyzed the data and participated in the discussion on views in the manuscript. All authors contributed to the article and approved the submitted version.

## Funding

This research was supported by Guizhou Forestry Scientific Research Project [Qianlin Kehe (202112)]; High level innovative talents training project of Guizhou Province (20154033); Guizhou Rural Industrial Revolution Dendrobium Industry Development Special Funds; He Chengyao National Medical Master Inheritance Studio.

## Conflict of interest

The study’s authors affirm that there were no financial or commercial ties that might be viewed as having a potential conflict of interest.

## Publisher’s note

All claims expressed in this article are solely those of the authors and do not necessarily represent those of their affiliated organizations, or those of the publisher, the editors and the reviewers. Any product that may be evaluated in this article, or claim that may be made by its manufacturer, is not guaranteed or endorsed by the publisher.

## Abbreviations

*D. officinale*: *Dendrobium officinale* Kimura et Migo
UHPLC-QQQ-MS/MS: ultrahigh performance liquid chromatography-triple quadrupole tandem mass spectrometry
TOPSIS: technique for order preference by similarity to ideal solution
LOD: limits of determination
LOQ: limits of quantification
*D*–: negative ideal solution distance
CI: composite score index
