# Supplementary material for: Simultaneous determination of nine phenolic compounds in imitation wild Dendrobium officinale samples using ultrahigh-performance liquid chromatography–tandem mass spectrometry
Source: Front Nutr. 2023 Apr 13;10:1129953. doi: 10.3389/fnut.2023.1129953 (PMC10134899; doi:10.3389/fnut.2023.1129953)
Supplement: Supplementary file 1 [file Table_1.docx]

**Table 6** weight of index

| item | Information entropy e | Information utility value d | Weight ( % ) |
| --- | --- | --- | --- |
| AWS | 0.8350 | 0.1650 | 5.804 |
| BYS | 0.3050 | 0.6950 | 24.43 |
| YPS | 0.8510 | 0.1490 | 5.226 |
| MXCS | 0.6750 | 0.3250 | 11.42 |
| BECS | 0.8340 | 0.1660 | 5.831 |
| HPS | 0.5940 | 0.4060 | 14.25 |
| YSLS | 0.4410 | 0.5590 | 19.64 |
| MXSG | 0.8150 | 0.1850 | 6.494 |
| YPG | 0.8030 | 0.1970 | 6.911 |

**Table 7** TOPSIS analysis intermediate value display

| item | positive ideal solution | negative ideal solution |
| --- | --- | --- |
| AWS | 0.6542 | 0 |
| BYS | 0.9966 | 0 |
| YPS | 0.6237 | 0 |
| MXCS | 0.9193 | 0.0004 |
| BECS | 0.6221 | 0 |
| HPS | 0.9273 | 0.0001 |
| YSLS | 0.9804 | 0.0003 |
| MXSG | 0.6287 | 0.0012 |
| YPG | 0.6953 | 0.0011 |
